# Retrospective Evaluation of Bone Turnover Markers in Serum for the Prediction of Metastases Development in Breast Cancer Patients: A Cohort Study

**DOI:** 10.3390/biomedicines12061201

**Published:** 2024-05-29

**Authors:** Mariz Kasoha, Sebastian Findeklee, Meletios P. Nigdelis, Gilda Schmidt, Erich-Franz Solomayer, Bashar Haj Hamoud

**Affiliations:** 1 Department of Gynecology, Obstetrics and Reproductive Medicine, University Medical School of Saarland, D-66421 Homburg, Germany; sebastian.findeklee@uks.eu (S.F.); meletios.nigdelis@uks.eu (M.P.N.); gilda.schmidt@uks.eu (G.S.); erich.solomayer@uks.eu (E.-F.S.); bashar.hajhamoud@uks.eu (B.H.H.); 2 Medizinische Versorgungszentrum, Göttingen, Kasseler Landstraße 25a, D-37081 Göttingen, Germany

**Keywords:** breast cancer, bone metastases, biomarkers, boner turnover

## Abstract

Background: Serum bone turnover markers might play a role in the prediction of the development of bone metastases in breast cancer (BC) patients. We conducted a retrospective cohort study to address the association of serum bone turnover markers with oncologic outcomes. Methods: We included 80 women with BC, who were operated on at the Department of Gynecology, Obstetrics and Reproductive Medicine, Homburg/Saar, Germany. Serum samples were obtained prior to surgery and were used for estimation of the concentration of tumor and bone turnover markers using enzyme-linked immunosorbent assay (ELISA) and radioimmunoassay (RIA). Results: At baseline, pyridinoline cross-linked carboxy-terminal telopeptide of type-1 collagen (ICTP) concentrations were higher in nodal positive vs. negative tumors (Mann–Whitney test *p* = 0.04). After a median follow-up of 79.4 months, 17 patients developed metastases, with 9 demonstrating, among other organs, osseous metastases. ICTP demonstrated the best area under the curve in the predection of osseous metastases in our cohort (AUC = 0.740, DeLong Test *p* = 0.005). Univariable Cox proportional hazard models failed to demonstrate significant associations between serum bone turnover markers and oncologic outcomes (progression-free survival, overall survival). Conclusions: Serum bone turnover markers (e.g., ICTP) were able to predict the development of osseous metastases but were not associated with oncologic outcomes. Further investigation and validation are required for the use of such markers in clinical practice.

## 1. Introduction

Breast cancer (BC) constitutes the most common malignancy among females, with an estimated incidence of 2,261,419 new cases per year according to the 2020 GLOBOCAN analysis, and is the second most common cause of cancer death in this group, mostly because of advanced/metastatic disease [1,2]. Nowadays, it is clearly understood that BC is clinically complex and biologically heterogenous, an aspect which is reflected in the classification of disease type, based on sex hormone receptor expression [estrogen receptor (ER), progesterone receptor (PR), and human epidermal growth factor receptor 2 (Her2)] [3].

Tumor metastatic potential is probably the most actively investigated aspect of BC pathobiology, even though common sites of metastases have been described; these include (with decreasing frequency) bone, axillary lymph nodes, liver, lung, and brain [3]. Osteotropism, the process through which tumor cells acquire molecular characteristics enabling them to enter blood circulation as disseminated tumor cells (DTCs) and attach to bone niches, has been attributed to the increased vascularity of the bone marrow along with pro-angiogenic growth factors and cytokines. In turn, DTCs have been hypothesized to remain in dormancy and/or give rise to osseous metastases, even though the exact mechanism leading to DTC activation is under investigation [4,5]. For example, based on preclinical data, Mercatalli et al. proposed that inhibition of epithelial growth factor receptor (EGFR) signaling may disturb the tumor-to-bone interaction [6]. Based on these findings, it is clearly understood that the clinical significance of utilizing biomarkers to identify patients at high risk of developing bone metastases lies in the potential of utilizing early-targeted bone therapies, such as denosumab [4].

Bone turnover markers (BTMs) are molecules reflecting osteoblastic and osteoclastic activity. These molecules include bone alkaline phosphatase (BAP), osteocalcin, and procollagen I N-propeptide (P1NP/PINP), illustrating bone formation, and degradation fragments of type I collagen (N- and C-telopeptides of type I collagen) and the enzyme tartrate-resistant acid phosphatase type 5 enzyme (TRAP5), reflecting osteoclastic activity. BTMs are traditionally measured using radioimmunoassay (RIA) and enzyme-linked immunoassay (ELISA) methodologies, or even automated processes involving chemiluminescence or electrochemiluminescence [7].

BTMs (anabolic and catabolic) have been studied in the setting of early and advanced BC [8]. Lumachi et al. demonstrated significant associations between baseline serum concentrations of bone-specific alkaline phosphatase, C-telopeptide of type I collagen (CTX), P1NP, and TRAP5 and the development of bone metastasis in a cohort of 297 patients with early luminal BC [9]. Similarly, Brown et al. evaluated baseline serum concentrations of bone remodeling markers in more than 800 patients from a randomized controlled trial of zolendronic acid in early BC. The authors demonstrated significant prognostic value in bone-specific recurrence for CTX, P1NP, and pyridinoline cross-linked carboxy-terminal telopeptide of type-1 collagen (ICTP) [10].

Recently, Shimoda et al. published a retrospective cohort study of 304 patients with resectable breast cancer evaluating serum levels of TRACP-5b. The authors demonstrated a statistically significant association between high TRACP-5b levels and a worse bone metastasis-free interval in nodal-positive tumors, further supporting the role of using BTMs as stratification markers for the development of bone metastases. Of course, the use of one single marker constitutes a significant disadvantage, which is encountered in the published literature [11].

Given the lack of clarity of association with further survival outcomes but also the common use of few markers, we conducted a retrospective cohort study evaluating the association of a large panel of BTMs with the development of metastases and survival outcomes.

## 2. Materials and Methods

### 2.1. Ethical Approval

Ethical approval was sought at the ethics committee of the state of Saarland (Reference number: 100/20, Approval date: 17 July 2020). The study was carried out in the Department of Gynecology, Obstetrics, and Reproductive Medicine at Saarland University Hospital in Germany, following the principles of the Helsinki Declaration. Written informed consent was obtained from all study participants prior to their taking part.

### 2.2. Participants and Clinicopathologic Data

This retrospective study involved 80 women with histologically confirmed early or advanced BC who underwent surgery at our department between 2010 and 2017. Diagnoses were based on the WHO classification in effect at the time of initial diagnosis.

Patients were identified in the prospective database of BC patients in our department. Participants in this study fulfilled the following inclusion criteria: blood sampling at the initial diagnosis of BC, patients with a follow-up time of more than 3 years, no current or history of other malignant disease or bone diseases, e.g., osteoporosis and Paget’s disease, no serious systemic diseases, proper adjuvant therapy was completed after operation in accordance with the German guideline at the time (adjuvant chemotherapy, endocrine therapy, trastuzumab targeted therapy in patients with positive Her2, and radiotherapy). Based on immunohistochemistry, tumors were divided into the four intrinsic subtypes [12]. Luminal A tumors included estrogen receptor (ER) and/or progesterone receptor (PR)-positive, tyrosine-protein kinase erbB-2 (Her2)-negative, and Ki-67 under 15%, luminal B tumors demonstrated the same receptor status, Ki-67 ≥ 15% [12]. The last two categories included Her2-positive tumors and triple-negative breast cancer (TNBC) tumors [12].

Demographic, clinical, pathologic, and follow-up data were collected from the pathology reports and medical records with the help of the System Analysis Program Development (SAP) software (Version Nr. SAP 7.70.5), the hospital’s internal system for storing patient data. The follow-up data, which included progression-free survival (PFS) and overall survival (OS), were stored in a prospective manner. PFS was defined as the time period from disease diagnosis to the first local or distant recurrence, measured in months. Overall survival (OS) was defined as the duration in months from the initial diagnosis of the disease to the time of death due to BC.

### 2.3. Serum Samples and Markers Analysis

Serum samples from the eighty patients who met the inclusion criteria were obtained from our department’s biobank. Blood samples were collected after peripheral venipuncture from patients on the day of surgery before anesthesia using serum gel monovette (S-Monovette Serum-Gel^®^, Sarstedt, Nümbrecht, Germany). Following centrifugation of the samples, the resulting supernatants were transferred into Eppendorf tubes and stored at −80 °C until analysis.

Serum concentrations of Dickkopf-1 protein 1 (Dkk1), sclerostin, receptor activator of nuclear factor kappa-Β ligand (RANKL), and osteoprotegerin (OPG) were measured in our research laboratory using Enzyme-linked Immunosorbent Assays (ELISA) kits from Biomedica^®^ Medizinprodukte GmbH, Vienna, Austria. The assays were conducted in accordance with the manufacturer’s protocol [13,14,15,16]. TRAP5, ICTP, and BAP serum concentrations were externally analyzed at Limbach Laboratory in Heidelberg, Germany using various immunoassays such as ELISA and radioimmunoassay (RIA). Cancer-antigen (Ca15-3) concentrations were collected from each patient’s medical record along with other clinical study data. Ca15-3 serum concentration is usually analyzed at our university hospital’s central laboratory using electrochemiluminescence immunoassay (ECLIA) technology from Roche^®^, Mannheim, Germany.

### 2.4. Statistical Analysis

#### 2.4.1. cBioPortal–TCGA Bioinformatic Analysis

**Figure 1:**
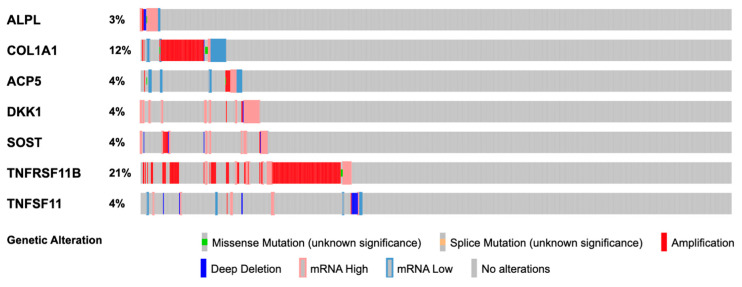
OncoPrint demonstrating gene alteration frequencies of the identified genes among the 816 patients reported by Ciriello as created in the cBioPortal [18,19,20,21].

We located the human genes (see Supplementary Table S1) encoding the bone turnover markers studied using the National Institutes of Health (NIH) Gene website [17]. A basic bioinformatic analysis was undertaken using the cBioPortal software (version 5.4.10) [18,19,20], using the Cancer Genome Atlas Program (TCGA) published whole-exome sequencing data of 817 patients with invasive carcinoma of the breast by Ciriello et al. [21]. Gene alteration frequencies of the identified genes along with the type of alterations were summarized in the OncoPrint (Figure 1).

#### 2.4.2. Statistical Analysis of Original Data

We tested the normality of continuous variables using the Shapiro–Wilk test. Continuous variables are presented as mean ± standard deviation, when normally distributed, and as median (range), when not normally distributed. Categorical variables are presented as absolute frequencies (percentages).

Given that all measured molecules were not normally distributed, group differences, in cases of variables with two groups, were controlled using the Mann–Whitney test. For variables with three or more groups, we used the Kruskal–Wallis test.

Cox proportional hazard models were fitted for progression (development of metastases or progression in case of baseline metastases) and death (overall survival) using the serum BTMs concentrations and other clinical parameters. Receiver operating curves (ROCs) were constructed by examining the association of serum markers and the development of metastases, and bone metastases among others, vs. controls (no metastases). For this analysis, we used the PPDA package of Jamovi, which only allowed for cases with completely reported data to be included. The statistical program Jamovi (2.3.21.0) was used for statistical analyses. Statistical significance was set at *p* < 0.05 unless otherwise specified. Missing data are reported along with summary estimates in the tables/text. We undertook no specific method to address this issue, such as imputation, given that there was no variable with >10% of data missing.

## 3. Results

### 3.1. In Silico Gene Alteration Analysis

Based on the cBioPortal analysis, a total of 305 (37%) patients demonstrated alterations of the genes of the studied BTMs. The most affected gene was *TNFRSF11B*, coding osteoprotegerin, followed by *COL1A1*, coding the pro-α1(I) chain of type I collagen. A variety of genetic alterations were observed, with amplification being the most common mechanism (See Figure 1).

### 3.2. Baseline Characteristics

The baseline characteristics of our sample are demonstrated in Table 1. We included 80 patients, of which 4 cases (5%) presented with metastatic disease at the time of first treatment (unknown at the time of blood sampling). Among these, three patients presented with osseous and one with liver metastases. Sixteen patients (20%) were pre- and perimenopausal, while the rest constituted postmenopausal BC cases.

A total of 17 other patients developed metastases during follow-up, in nine of whom the bones were affected. Median follow-up exceeded 5 years.

### 3.3. Subgroup Differences

Table 2 demonstrates variable differences, including serum BTMs concentrations, between the four molecular BC subgroups. In our cohort, we were able to demonstrate significant differences in age (Kruskal–Wallis test, *p* = 0.03) and Ki67-Score (Kruskal–Wallis test, *p* < 0.001). No statistically significant differences in the concentration of BTMs were demonstrated.

Differences in BTMs concentrations based on clinical characteristics other than molecular subtype are illustrated in Table 3. Compared with ductal cancer, patients with invasive lobular cancer had higher concentrations of Dkk1 [median (range): 1424 pg/mL (672–2565 pg/mL) vs. 1120 pg/mL (419–2727 pg/mL), Mann–Whitney test *p* = 0.02] and ICTP [3.5 μg/L (1.8–17.0 μg/L) vs. 2.9 μg/L (1.1–11.0 μg/L), Mann–Whitney test *p* = 0.005]. Similarly, ICTP concentrations were significantly higher in nodal positive compared with nodal negative tumors [3.4 μg/L (1.9–17.0 μg/L) vs. 2.9 μg/L (1.1–11.0 μg/L), Mann–Whitney test *p* = 0.04].

We observed a marginal trend, yet not statistically significant, between the concentration of ICTP and the development of metastases, as these patients demonstrated higher concentrations compared to those not developing metastasis [3.2 μg/L (2.4–10.0 μg/L) vs. 2.9 μg/L (1.1–17.0 μg/L), Mann–Whitney test *p* = 0.05]. No statistically significant differences were observed in concentrations of serum BTMs between patients developing metastases including osseous ones vs. those with metastases not involving the bone.

### 3.4. Univariable Cox Proportional Hazards Model

Table 4 demonstrates the univariable Cox regression models for PFS (development of metastases and progression for patients presenting with metastases at baseline) and OS. Statistically significant associations were demonstrated between the T3-4 stage (compared with the T1 stage, *p* = 0.02), nodal positive tumors (*p* = 0.002), and Ki67 ≥ 15% (*p* = 0.03) and PFS. In terms of OS, significant associations were demonstrated for Stage T3-4 (*p* = 0.03) and Ki67 ≥ 15% (*p* = 0.04). Serum concentrations of different BTMs (division in groups according to the median value of each variable) were not significantly associated with PFS or OS.

### 3.5. Receiver Operating Curves (ROCs)

Assessing the performance of serum markers to predict metastases development we constructed ROCs.

#### 3.5.1. All Types of Metastases (Figure 2)

Data from 68 patients were used (complete reporting and patients who did not present with metastases at baseline). Concentrations of ICTP demonstrated the best area under the curve (AUC) 0.685 with specificity 88.68%, 90.57%, and 92.45%, and sensitivity 46.67%, 46.67%, and 40% when cut points of 3.8 μg/L, 3.9 μg/L, and 4.3 μg/L were considered, respectively. Nonetheless, the DeLong test did not demonstrate any statistically significant differences between the AUC of different markers (*p* = 0.58).

**Figure 2:**
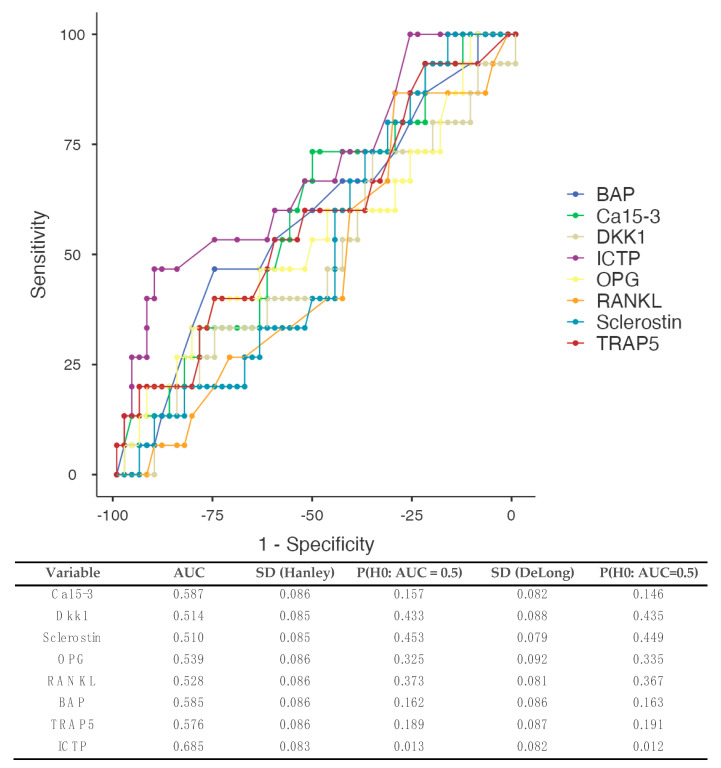
Receiver operating curves (ROCs) and the prediction of metastases development based on different serum BTMs. Patients presenting with incomplete data and/or metastases were excluded from the analysis (*n* = 12). Estimated areas under the curve (AUCs) are demonstrated in the table below the figure. DeLong’s test demonstrated no significant differences between the different curves (overall *p*-value = 0.58). AUC: area under the curve, BAP: bone alkaline phosphatase, Dkk1: Dickkopf-1, ICTP: carboxyterminal telopeptide of type I collagen, OPG: osteoprotegerin, RANKL: receptor activator of nuclear factor kappa beta, TRAP5: tartrate-resistant acid phosphatase 5.

#### 3.5.2. Patients Developing Bone Metastases (Figure 3)

A total of 62 patients contributed to the analysis. ICTP demonstrated the best AUC of 0.740, while BAP and Ca15-3 were the next best with AUCs of 0.680 and 0.671, respectively. The DeLong test demonstrated statistically significant differences (*p* = 0.005), with ICTP demonstrating the best performance.

**Figure 3:**
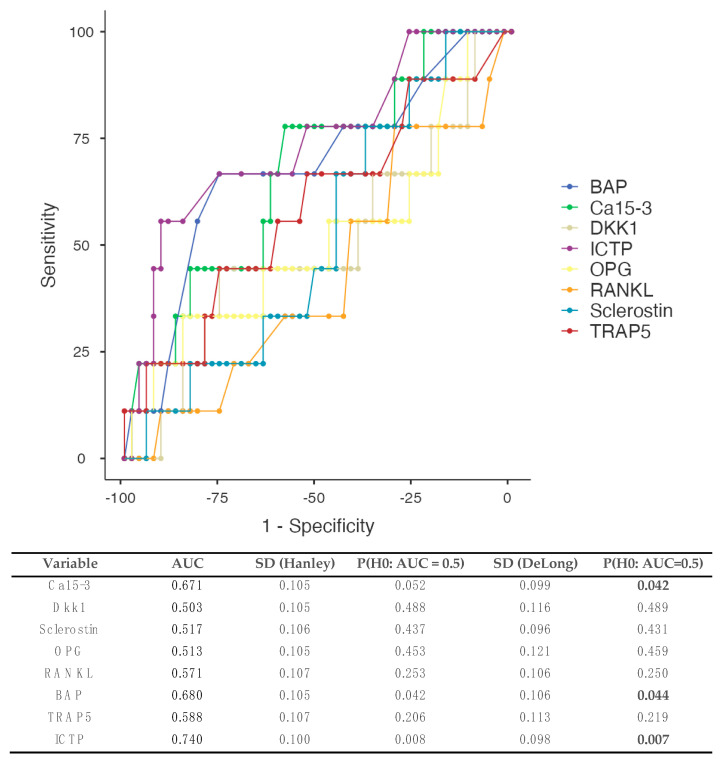
Receiver operating curves (ROCs) and the prediction of metastases development involving, among others, the bones, based on different serum BTMs. Patients presenting with incomplete data, metastases at diagnosis, and/or development of metastases not including the bones were excluded from the analysis (*n* = 18). Estimated areas under the curve (AUCs) are demonstrated in the table below the figure. DeLong’s test demonstrated statistically significant differences between the different curves, with BAP and ICTP demonstrating higher AUCs (overall *p*-value = 0.005). AUC: area under the curve, BAP: bone alkaline phosphatase, Dkk1: Dickkopf-1, ICTP: carboxyterminal telopeptide of type I collagen, OPG: osteoprotegerin, RANKL: receptor activator of nuclear factor kappa beta, TRAP5: tartrate-resistant acid phosphatase 5.

## 4. Discussion

In this cohort study, we demonstrated the importance of BTMs in early BC as demonstrated by genetic alterations of BTM genes in BC through a simple bioinformatic analysis. Furthermore, we were not able to demonstrate statistically significant differences between tested BTMs and the development of metastases among BC patients. Lobular histology and nodal-positive disease were associated with higher concentrations of ICTP compared with ductal histology and nodal-negative disease, respectively. We demonstrated no differences in bone marker concentration and different molecular subtypes. PFD was associated with classical clinicopathologic parameters (Stage T3-4, nodal positive disease, Ki67 ≥ 15%), while OS was associated with Stage T3-4 and Ki67 ≥ 15%; different groups of serum concentrations were not significantly associated with PFD or OS.

ICTP demonstrated the highest AUC 0.685 for the prediction of metastases, even though there was no significant difference among the ROCs of different markers. In cases of patients developing, among others, bone metastases, BAP and ICTP performed significantly better compared with the rest of the bone markers (AUC 0.68, and 0.74, DeLong test *p* = 0.005).

In the last decades, type I collagen and its fragments have gained interest in tumor biology, as studies have demonstrated its role in cellular proliferation, epithelial-mesenchymal transition, cellular invasion, development of metastases, and efficacy of anti-cancer treatments [22,23]. Specifically for BC, Liu et al. demonstrated an upregulation in the expression of collagen type I alpha 1 (COL1A1) in BC cells, which was associated with poorer OS. The authors concluded that this biological process might constitute a potential treatment target, which remains to be investigated [24].

In terms of ICTP and other collagen fragments, early BC studies have demonstrated unequivocal findings, requiring critical assessment and further research. In a large prospective study, elevated preoperative concentrations of serum ICTP were associated with increased BC-specific survival in luminal B tumors, and local relapse-free survival in TNBC [25]. In contrast to this study, Imamura et al. found that postmenopausal patients with elevated concentrations of serum ICTP had a poorer relapse-free survival rate [26].

Apart from survival outcomes, the development of bone metastases has also been studied. As mentioned above, Shimoda et al. demonstrated a possible role of high TRACP-5b levels in the prediction of bone metastasis development among nodal-positive resectable BC cases [11]. In a subanalysis of the AZURE (BIG01/04) trial, involving early BC cases, Brown et al. demonstrated significant prognostic ability for bone recurrence for P1NP, CTX, and 1-CTP, as Zuo et al. did in the case of P1NP and CTX [10,27]. Regarding our study, we were not able to demonstrate a significant association between ICTP and other BTMs and survival outcomes. Still, ICTP constituted the marker with the highest AUC in terms of development of metastases and bone metastases among our cohort.

An interesting aspect, which is suggested by these findings, is the potential of BTMs to stratify patients at a high risk of (bone) metastatic disease and govern further treatments in the adjuvant setting to increase bone-metastasis-free survival; a comprehensive list of biomarkers is provided by Wang et al. [28]. Interestingly, stronger evidence of this notion has arisen in the context of advanced disease [29,30,31]. D’Oronzo and colleagues recently studied 47 patients with bone metastatic BC who underwent BTM measurements and dual-energy X-ray absorptiometry (DXA) before bisphosphonate treatment. The authors showed that OPG levels < 5.2 pmol/L were associated with an increased risk of progression. Skeletal-related events were also significantly associated with lower concentrations of osteocalcin, OPG, lower lumbar T-Score, and femur bone mineral density at baseline [30]. Another prospective study of patients with bone metastases (various tumors including BC) by Ibrahim et al. demonstrated that RANKL transcription levels were the best marker of the response of bone metastases during treatment with zoledronic acid [31]. In a similar sense to metastatic disease, similar studies should be attempted in the adjuvant setting (decreasing the risk of bone metastases using therapies based on BTM concentration changes), even though such attempts might be more copious from a methodologic perspective (long follow-up, resources).

Apart from collagen fragments, our study also focused on Wnt-signaling pathway inhibitors, namely Dkk1 and sclerostin. These two proteins inhibit the canonical Wnt β-catenin pathway by binding to low-density lipoprotein receptor-related protein 5/6 (LRP5/6) and Frizzled protein, which, in turn, leads to a predominance of osteoclastic activity (bone resorption) [32,33]. Many research groups, including ours, have demonstrated that BC is associated with an aberrant expression of Dkk1—a review of mechanisms can be found in the review article by Kasoha et al. [34,35,36]. More specifically, we have demonstrated that patients with early primary BC had increased concentrations compared with healthy controls, while patients with bone metastases had the highest concentrations among the three groups [36]. Even though Dkk1 concentrations were significantly higher among lobular BC cases, we were unable to demonstrate a significant correlation between serum concentrations of Dkk1 and sclerostin and the development of metastases in the current study. This observation may partially support the finding by Geyer et al., who showed that lobular carcinomas lack β-catenin expression on the cellular membrane, cytoplasm, and the nucleus, as also described by [37,38]. More evidence is required to elucidate this aspect.

The tumor marker Ca15-3 was also studied in our cohort. We found that serum concentrations of Ca15-3 demonstrated an AUC of 0.587 (for all metastases) and 0.671 when patients without bone metastases were excluded from the analyses. In a retrospective analysis of 389 BC patients, Zhang and colleagues demonstrated predictive accuracy for distant metastases for Ca15–3 (AUC = 0.821). Of note, the combination of a panel of tumor markers [polypeptide specific antigen (TPS), carcinoembryonic antigen (CEA), and Ca125] was not superior to Ca15-3 [39].

Our study presents both strengths and limitations. As far as strengths are concerned, we evaluated a panel of various BTMs in the serum of BC patients (both anabolic and catabolic pathways were involved). Furthermore, median follow-up exceeded 5 years (79.4 months), which provides strength in capturing oncologic events despite the limited number of participants in the study. As far as limitations are concerned, given the absence of serial serum measurements, the exact longitudinal changes of bone turnover markers in patients developing metastases remain uncaptured. This might have clinical significance (see above), but could, unfortunately, not be tackled in a retrospective study such as this. Assessing further markers, BTMs concentrations or even genomic or proteomic profiling data, in tissues other than serum (e.g., BC tissue) might have yielded more accurate results, reflecting tumor metastatic potential. Furthermore, assessing patients developing metastases of one organ type is also an important aspect, requiring much larger sample sizes.

Our study provided evidence on the possibility of using a baseline blood sample at diagnosis of BC for patient stratification regarding the risk of developing (bone) metastases. Longitudinal measurements along with larger population samples are required to draw safer conclusions and establish these biomarkers in clinical practice. Future studies need to evaluate these molecules on an OMIC level or based on a systems approach for a better understating of their biological role in the development of bone metastases, but also for disease monitoring in the adjuvant and metastatic treatment setting.

## Figures and Tables

**Table 1:** Baseline characteristics of the cohort (*n* = 80). For continuous variables, we used median (range), while qualitative variables are summarized as absolute frequency (percentage).

Variable	Value	Missing Data
Age (years)	62.4 (46.1–84.2)	-
BMI (kg/m^2^)	25.7 (18.7–32.7)	-
Pre-/perimenopausal	16 (20%)	-
Histological subtypes	-	
Ductal/lobular	59 (73.8%)/21 (26.3%)	
Molecular subtypes	-	
Luminal A	39 (48.8%)	
Luminal B	30 (37.5%)	
Her2 positive	5 (6.3%)	
Triple negative	6 (7.5%)	
T-Stage	-	
T1/T2/T3-4	50 (62.5 %)/27 (33.8 %)/3 (3.8 %)	
N-Stage	-	
N0/N1/N2-3	54 (67.5%)/22 (27.5%)/4 (5%)	
Presence of metastases	4 (5%)	-
G-Stage		-
G1/G2/G3	6 (7.5%)/62 (77.5%)/12 (15%)	
Ki-67 (%)	10 (2–70)	1 (1.3%)
Follow-up (months)	79.4 (15.5–161)	
Ca15-3 (U/mL)	19.3 (6.30–151)	8 (10%)
Dkk1 (pg/mL)	1249 (419–2727)	-
Sclerostin (pg/L)	709 (182–3818)	-
OPG (pg/mL)	84.4 (15.2–202)	-
RANKL (pg/mL)	2.4 (0.4–7.6)	-
OPG/RANKL	35.5 (4.84–305)	-
BAP (μg/L)	16. (6.2–33.0)	-
TRAP5 (U/L)	2.95 (1.5–21.0)	-
ICTP (μg/L)	3.0 (1.1–17.0)	-

BAP: bone alkaline phosphatase, BMI: body mass index, Dkk1: Dickkopf-1, ICTP: carboxyterminal telopeptide of type I collagen, OPG: osteoprotegerin, RANKL: receptor activator of nuclear factor kappa beta, TRAP5: tartrate-resistant acid phosphatase 5.

**Table 2:** Comparison of clinical characteristics and serum markers between different molecular subtypes in the cohort. Variables are presented as median (range). Statistically significant results are depicted in bold.

Variable	Luminal A (*n* = 39)	Luminal B (*n* = 30)	TNBC (*n* = 6)	Her2neu Positive (*n* = 5)	*p*-Value ^1^
Age (years)	58.7 (46.1–80.2)	67.7 (49.6–82.7)	66.2 (49.5–84.2)	69.1 (51.1–77.1)	**0.03**
BMI (kg/m^2^)	25.6 (18.7–31.9)	26.3 (21.1–32.7)	24.9 (19.8–27.8)	24.7 (23.0–27.5)	0.24
Ki67 (%)	10 (2–15)	25 (15–50)	40 (15–70)	10 (10–20)	**<0.001**
Ca15-3 (U/mL)	17.8 (7.2–54.0)	22.6 (7.6–37.6)	28.5 (13.0–37.8)	21.4 (6.3–151)	0.43
Dkk1 (pg/mL)	1311 (419–2727)	1151 (460–2113)	1063 (652–1935)	1335 (1110–1679)	0.74
Sclerostin (pg/L)	655 (182–1834)	761 (189–3818)	770 (468–2043)	784 (607–952)	0.79
OPG (pg/mL)	86.0 (15.2–177)	76.1 (23.4–187)	107 (83.8–202)	75.4 (24.0–144)	0.11
RANKL (pg/mL)	2.4 (0.6–7.6)	2.3 (0.4–7.0)	3.0 (0.6–3.4)	2.0 (0.8–4.6)	0.98
OPG/RANKL	39.7 (4.8–177)	35.1 (7.1–305)	46.0 (25.2–200)	33.2 (10.9–180)	0.77
BAP (μg/L)	14.0 (6.2–27.0)	17.0 (8.2–33.0)	19.0 (12.0–32.0)	19.0 (14.0–25.0)	0.09
TRAP5 (U/L)	2.8 (1.5–6.0)	3.0 (1.6–21.0)	4.1 (1.7–6.7)	2.9 (2.0–5.7)	0.32
ICTP (μg/L)	2.9 (1.2–17.0)	3.1 (1.1–10.0)	3.4 (2.8–11.0)	3.6 (3.4–4.3)	0.08

BAP: bone alkaline phosphatase, BMI: body mass index, Dkk1: Dickkopf-1, ICTP: carboxyterminal telopeptide of type I collagen, OPG: osteoprotegerin, RANKL: receptor activator of nuclear factor kappa beta, TRAP5: tartrate-resistant acid phosphatase 5. ^1^ *p*-values correspond to those of Kruskal–Wallis test.

**Table 3:** Comparison of serum BTMs between various clinial subgroups in the cohort. Variables are presented as median (range). In cases of two subgroups, statistical comparisons were made using the Mann–Whitney test, whereas in cases of >2 groups we used the Kruskall–Wallis test. Statistically significant results are depicted in bold.

Variable	Ca15-3	*p*	Dkk1	*p*	Sclerostin	*p*	OPG	*p*	RANKL	*p*	OPG/RANKL	*p*	BAP	*p*	TRAP5	*p*	ICTP	*p*
**Age**		0.06		0.61		0.56		0.64		0.45		0.49		0.60		0.94		0.90
≤55 years old	17.8 (6.3–54.0)		1254 (553–2429)		789 (189–1434)		83.0 (24.0–130)		2.6 (0.6–7.0)		28.1 (4.8–130)		16.0 (6.2–27.0)		3.1 (1.5–5.5)		3.2 (1.1–4.7)	
>55 years old	21.4 (7.2–151)		1235 (419–2727)		695 (182–3818)		86.0 (15.2–202)		2.4 (0.4–7.6)		35.8 (4.8–305)		16.0 (8.1–33.0)		2.9 (1.6–21.0)		3.0 (1.2–17.0)	
**Menopausal status**		0.05		0.22		0.17		0.93		0.63		0.77		0.48		0.51		0.19
Pre-/perimenopausal	17.8 (6.3–54.0)		1379 (553–2429)		819 (314–1225)		83.4 (24.0–130)		2.4 (0.6–7.0)		37.1 (4.8–130)		17.0 (8.9–23.0)		2.8 (1.7–5.5)		3.4 (1.5–17.0)	
Postmenopausal	19.6 (7.2–151)		1219 (419–2727)		670 (182–3818)		85.0 (15.2–202)		2.4 (0.4–7.6)		35.5 (5.1–305)		16.0 (6.2–33.0)		3.0 (1.5–21.0)		3.0 (1.1–11.0)	
**Histology**		0.66		**0.02**		0.09		0.14		0.41		0.35		0.29		0.71		**0.005**
Invasive ductal	19.2 (7.2–54.0)		1120 (419–2727)		655 (182–3818)		83.4 (15.2–202)		2.4 (0.6–7.6)		35.8 (4.8–233)		16.0 (6.2–33.0)		3.0 (1.5–6.7)		2.9 (1.1–11.0)	
Invasive lobular	22.6 (6.3–151)		1424 (672–2565)		751 (339–1823)		86.2 (24.0–176)		2.4 (0.4–4.6)		34.8 (10.9–305)		16.0 (8.2–33.0)		2.8 (1.7–21.0)		3.5 (1.8–17.0)	
T-Stage		0.08		0.68		0.05		0.87		0.43		0.92		0.07		0.14		0.28
T1	17.8 (7.2–37.8)		1294 (423–2727)		652 (182–2043)		85.0 (25.4–202)		2.5 (0.6–7.6)		39.4 (4.84–294)		14.5 (6.2–33.0)		2.9 (1.5–6.7)		3.0 (1.1–17.0)	
T2	20.2 (6.30–151)		1102 (419–2113)		859 (198–3818)		83.0 (15.2–187)		2.0 (0.4–5.6)		34.6 (5.07–305)		19.0 (9.0–33.0)		3.1 (1.6–21.0)		3.2 (2.0–10.0)	
T3-4	30.6 (29.5–31.8)		1341 (881–1638)		345 (339–827)		86.2 (46.0–103)		3.4 (1.2–5.6)		30.4 (8.21–71.8)		16.0 (12.0–17.0)		4.1 (3.6–5.8)		3.1 (2.5–6.7)	
**N-Stage**		0.07		0.87		0.50		0.65		0.15		0.29		0.40		0.60		**0.04**
N negative	18.7 (6.3–37.8)		1271 (423–2727)		701 (182–2043)		85.4 (23.4–202)		2.5 (0.6–7.6)		34.3 (4.8–294)		16.0 (8.1–33.0)		2.9 (1.5–6.7)		2.9 (1.1–11.0)	
N positive	21.4 (12.8–151)		1208 (419–2113)		711 (198–3818)		83.4 (15.2–187)		2.0 (0.4–5.6)		35.6 (5.1–305)		16.5 (6.2–33.0)		3.2 (1.6–21)		3.4 (1.9–17.0)	
**G-Status**		0.55		0.36		0.37		0.62		0.53		0.41		0.28		0.76		0.60
G1	17.8 (12.3–28.7)		1336 (423–2727)		853 (314–1595)		67.0 (25.4–177)		3.1 (1.0–7.6)		18.4 (4.8–177)		12.5 (8.9–17.0)		2.9 (2.1–4.2)		3.2 (2.0–4.5)	
G2	19.2 (6.30–54.0)		1248 (419–2565)		748 (182–3818)		85.0 (15.2–202)		2.4 (0.4–7.0)		38.4 (4.8–305)		16.0 (6.2–33.0)		3.0 (1.5–21.0)		2.9 (1.1–17.0)	
G3	22.1 (8.00–151)		935 (512–1708)		651 (332–859)		84.8 (23.4–120)		2.2 (0.6–6.6)		31.6 (10.2–200)		19.0 (8.1–25.0)		3.0 (1.6–5.8)		3.3 (2.4–4.5)	
**Development of** **metastases ^1^**		0.31		0.65		0.98		0.76		0.82		0.86		0.30		0.15		**0.053**
No metastases	18.1 (6.3–54.0)		1308 (423–2727)		718 (182–3818)		85.0 (23.4–202)		2.4 (0.4–7.6)		35.8 (4.8–305)		16.0 (6.2–33.0)		2.9 (1.5–6.7)		2.9 (1.1–17.0)	
Developed metastases	20.2 (10.7–38.9)		1181 (419–2016)		709 (225–1823)		85.8 (15.2–187)		2.0 (0.6–5.6)		37.6 (5.1–233)		17.0 (9.9–33.0)		3.4 (1.7–21.0)		3.20 (2.4–10.0)	
**Development of bone metastases ^2^**		0.08		0.74		0.61		1.00		0.23		0.42		0.07		0.89		0.44
Bone	18.9 (10.7–28.5)		1173 (419–1935)		681 (225–1430)		94.5 (15.2–135)		2.7 (1.6–4.0)		31.3 (5.1–61.6)		13.5 (9.9–20.0)		3.3 (2.3–6.0)		3.1 (2.4–6.8)	
Other than bone	22.9 (13.0–38.9)		1181 (652–2016)		714 (345–1823)		83.8 (46.0–187)		2.0 (0.6–5.6)		39.1 (8.2–233)		21.0 (11.0–33.0)		3.4 (1.7–21.0)		3.9 (2.4–10)	

BAP: bone alkaline phosphatase, Dkk1: Dickkopf-1, ICTP: carboxyterminal telopeptide of type I collagen, OPG: osteoprotegerin, RANKL: receptor activator of nuclear factor kappa beta, TRAP5: tartrate-resistant acid phosphatase 5. ^1^ patients presenting with metastases at baseline were excluded. ^2^ only patients developing metastases were compared.

**Table 4:** Univariable proportional hazard models (Cox models). The left side of the table refers to PFS (development of metastases and progression for patients presenting with metastases at baseline), while the right side of the table refers to OS. Statistically significant results are depicted in bold.

Variable	Progression		Death	
	HR (95% CI)	*p*-Value	HR (95% CI)	*p*-Value
Histology				
Ductal	-	-	-	-
Lobular	0.68 (0.22–2.07)	*p* = 0.50	0.94 (0.33–2.70)	*p* = 0.91
T-Stage				
T1	-	-	-	-
T2	2.47 (0.95–6.41)	*p* = 0.06	2.20 (0.80–6.08)	*p* = 0.13
T3–4	6.61 (1.39–31.37)	***p* = 0.02**	5.66 (1.17–27.33)	***p* = 0.03**
N-Stage				
N negative	-	-	-	-
N positive	4.39 (1.73–11.16)	***p* = 0.002**	2.60 (1.00–6.73)	*p* = 0.05
Ki67				
Ki67 < 15%	-	-	-	-
Ki67 ≥ 15%	3.00 (1.11–8.09)	***p* = 0.03**	3.06 (1.06–8.87)	***p* = 0.04**
Ca15-3 (median 26.4 U/mL)				
<26.4 U/mL	-	-	-	-
≥26.4 U/mL	1.27 (0.45–3.62)	*p* = 0.65	1.38 (0.47–4.04)	*p* = 0.56
Dkk1 (median 1249 pg/mL)				
<1249 pg/mL	-	-	-	-
≥1249 pg/mL	0.58 (0.23–1.47)	*p* = 0.25	0.74 (0.28–1.95)	*p* = 0.54
Sclerostin (median 709 pg/L)				
<709 pg/L			-	-
≥709 pg/L	0.80 (0.32–1.97)	*p* = 0.63	1.43 (0.54–3.76)	*p* = 0.47
OPG (median 84.4 pg/mL)				
<84.4 pg/L	-	-	-	-
≥84.4 pg/L	1.28 (0.51–3.18)	*p* = 0.60	1.48 (0.56–3.90)	*p* = 0.43
RANKL (median 2.4 pg/mL)				
<2.4 pg/mL	-	-	-	-
≥2.4 pg/mL	1.11 (0.44–2.82)	*p* = 0.83	1.31 (0.46–3.72)	*p* = 0.62
BAP (median 16 μg/L)				
<16 μg/L	-	-	-	-
≥16 μg/L	1.14 (0.46–2.82)	*p* = 0.79	0.90 (0.35–2.34)	*p* = 0.83
TRAP5 (median 2.95 U/L)				
<2.95 U/L	-	-	-	-
≥2.95 U/L	1.59 (0.62–4.03)	*p* = 0.33	1.42 (0.54–3.73)	*p* = 0.48
ICTP (median 3.0 μg/L)				
<3.0 μg/L	-	-	-	-
≥3.0 μg/L	2.02 (0.77–5.31)	0.15	1.58 (0.58–4.27)	*p* = 0.37

BAP: bone alkaline phosphatase, Dkk1: Dickkopf-1, ICTP: carboxyterminal telopeptide of type I collagen, OPG: osteoprotegerin, RANKL: receptor activator of nuclear factor kappa beta, TRAP5: tartrate-resistant acid phosphatase 5.

## Data Availability

Study data can be made available after contacting the corresponding author.

## Supplementary Materials

The following supporting information can be downloaded at: https://www.mdpi.com/article/10.3390/biomedicines12061201/s1, Supplementary Table S1. Summary of human genes for studied proteins along with frequencies of gene alterations in the TCGA cohort published by Ciriello et al. [21] (see Section 2.4.1).

## References

[1] Sung H., Ferlay J., Siegel R.L., Laversanne M., Soerjomataram I., Jemal A., Bray F. (2021). Global Cancer Statistics 2020: GLOBOCAN Estimates of Incidence and Mortality Worldwide for 36 Cancers in 185 Countries. CA Cancer J. Clin..

[2] Giaquinto A.N., Sung H., Miller K.D., Kramer J.L., Newman L.A., Minihan A., Jemal A., Siegel R.L. (2022). Breast Cancer Statistics, 2022. CA Cancer J. Clin..

[3] Harbeck N., Penault-Llorca F., Cortes J., Gnant M., Houssami N., Poortmans P., Ruddy K., Tsang J., Cardoso F. (2019). Breast cancer. Nat. Rev. Dis. Primers.

[4] Iuliani M., Simonetti S., Ribelli G., Napolitano A., Pantano F., Vincenzi B., Tonini G., Santini D. (2020). Current and Emerging Biomarkers Predicting Bone Metastasis Development. Front. Oncol..

[5] Braun S., Vogl F.D., Naume B., Janni W., Osborne M.P., Coombes R.C., Schlimok G., Diel I.J., Gerber B., Gebauer G. (2005). A pooled analysis of bone marrow micrometastasis in breast cancer. N. Engl. J. Med..

[6] Mercatali L., La Manna F., Miserocchi G., Liverani C., De Vita A., Spadazzi C., Bongiovanni A., Recine F., Amadori D., Ghetti M. (2017). Tumor-Stroma Crosstalk in Bone Tissue: The Osteoclastogenic Potential of a Breast Cancer Cell Line in a Co-Culture System and the Role of EGFR Inhibition. Int. J. Mol. Sci..

[7] Schini M., Vilaca T., Gossiel F., Salam S., Eastell R. (2023). Bone Turnover Markers: Basic Biology to Clinical Applications. Endocr. Rev..

[8] Ferreira A., Alho I., Casimiro S., Costa L. (2015). Bone remodeling markers and bone metastases: From cancer research to clinical implications. BoneKEy Rep..

[9] Lumachi F., Basso S.M., Camozzi V., Tozzoli R., Spaziante R., Ermani M. (2016). Bone turnover markers in women with early stage breast cancer who developed bone metastases. A prospective study with multivariate logistic regression analysis of accuracy. Clin. Chim. Acta.

[10] Brown J., Rathbone E., Hinsley S., Gregory W., Gossiel F., Marshall H., Burkinshaw R., Shulver H., Thandar H., Bertelli G. (2018). Associations between Serum Bone Biomarkers in Early Breast Cancer and Development of Bone Metastasis: Results from the AZURE (BIG01/04) Trial. J. Natl. Cancer Inst..

[11] Shimoda M., Sato Y., Abe K., Masunaga N., Tsukabe M., Yoshinami T., Sota Y., Miyake T., Tanei T., Shimazu K. (2024). Prognostic value of serum tartrate-resistant acid phosphatase-5b for bone metastasis in patients with resectable breast cancer. Oncol. Lett. Oncol. Lett..

[12] (2021). S3-Leitlinie Früherkennung, Diagnostik, Therapie und Nachsorge des Mammakarzinoms. https://register.awmf.org/de/leitlinien/detail/032-045OL.

[13] Biomedica Protocol Booklet: DKK-1. https://www.bmgrp.com/wp-content/uploads/2022/05/BI-20413-DKK-1-ELISA-IFU-220524.pdf.

[14] Biomedica Protocol Booklet: Sclerostin. https://www.bmgrp.com/wp-content/uploads/2022/05/BI-20492-Sclerostin-ELISA-IFU-220524.pdf.

[15] Biomedica Protocol Booklet: Osteoprotegerin. https://www.bmgrp.com/wp-content/uploads/2023/04/BI-20403-OPG-ELISA-IFU-230323.pdf.

[16] Biomedica Protocol Booklet: Free soluble RANKL. https://www.bmgrp.com/wp-content/uploads/2022/05/BI-20462-free-soluble-RANKL-ELISA-IFU-220524.pdf.

[17] Home—Gene—NCBI. https://www.ncbi.nlm.nih.gov/gene.

[18] Cerami E., Gao J., Dogrusoz U., Gross B.E., Sumer S.O., Aksoy B.A., Jacobsen A., Byrne C.J., Heuer M.L., Larsson E. (2012). The cBio cancer genomics portal: An open platform for exploring multidimensional cancer genomics data. Cancer Discov..

[19] Gao J., Aksoy B.A., Dogrusoz U., Dresdner G., Gross B.E., Sumer S.O., Sun Y., Jacobsen A., Sinha R., Larsson E. (2013). Integrative analysis of complex cancer genomics and clinical profiles using the cBioPortal. Sci. Signal..

[20] de Bruijn I., Kundra R., Mastrogiacomo B., Tran T.N., Sikina L., Mazor T., Li X., Ochoa A., Zhao G., Lai B. (2023). Analysis and Visualization of Longitudinal Genomic and Clinical Data from the AACR Project GENIE Biopharma Collaborative in cBioPortal. Cancer Res..

[21] Ciriello G., Gatza M.L., Beck A.H., Wilkerson M.D., Rhie S.K., Pastore A., Zhang H., McLellan M., Yau C., Kandoth C. (2015). Comprehensive Molecular Portraits of Invasive Lobular Breast Cancer. Cell.

[22] Shi R., Zhang Z., Zhu A., Xiong X., Zhang J., Xu J., Sy M., Li C. (2022). Targeting type I collagen for cancer treatment. Int. J. Cancer.

[23] Xu S., Xu H., Wang W., Li S., Li H., Li T., Zhang W., Yu X., Liu L. (2019). The role of collagen in cancer: From bench to bedside. J. Transl. Med..

[24] Liu J., Shen J.-X., Wu H.-T., Li X.-L., Wen X.-F., Du C.-W., Zhang G.-J. (2018). Collagen 1A1 (COL1A1) promotes metastasis of breast cancer and is a potential therapeutic target. Discov. Med..

[25] Jääskeläinen A., Jukkola A., Risteli J., Haapasaari K.-M., Karihtala P. (2019). Elevated preoperative serum levels of collagen I carboxyterminal telopeptide predict better outcome in early-stage luminal-B-like (HER2-negative) and triple-negative subtypes of breast cancer. Tumor Biol..

[26] Imamura M., Nishimukai A., Higuchi T., Ozawa H., Yanai A., Miyagawa Y., Murase K., Sakita I., Hatada T., Takatsuka Y. (2015). High levels at baseline of serum pyridinoline crosslinked carboxyterminal telopeptide of type I collagen are associated with worse prognosis for breast cancer patients. Breast Cancer Res. Treat..

[27] Zuo C.-T., Yin D.-C., Fan H.-X., Lin M., Meng Z., Xin G.-W., Zhang Y.-C., Cheng L. (2019). Study on diagnostic value of P1NP and β-CTX in bone metastasis of patients with breast cancer and the correlation between them. Eur. Rev. Med. Pharmacol. Sci..

[28] Wang S., Wu W., Lin X., Zhang K.M., Wu Q., Luo M., Zhou J. (2023). Predictive and prognostic biomarkers of bone metastasis in breast cancer: Current status and future directions. Cell Biosci..

[29] Galliera E., Massaccesi L., de Benedettis E., Longhi E., de Toma D., Romanelli M.M.C., Banfi G. (2020). Longitudinal evaluation of Wnt inhibitors and comparison with others serum osteoimmunological biomarkers in osteolytic bone metastasis. J. Leukoc. Biol..

[30] D’oronzo S., Cives M., Lauricella E., Stucci S., Centonza A., Gentile M., Ostuni C., Porta C. (2024). Assessment of bone turnover markers and DXA parameters to predict bone metastasis progression during zoledronate treatment: A single-center experience. Clin. Exp. Med..

[31] Ibrahim T., Ricci M., Scarpi E., Bongiovanni A., Ricci R., Riva N., Liverani C., De Vita A., La Manna F., Oboldi D. (2016). RANKL: A promising circulating marker for bone metastasis response. Oncol. Lett..

[32] Dincel A.S., Jørgensen N.R. (2022). New Emerging Biomarkers for Bone Disease: Sclerostin and Dickkopf-1 (DKK1). Calcif. Tissue Int..

[33] Clevers H., Nusse R. (2012). Wnt/β-Catenin Signaling and Disease. Cell.

[34] El-Mahdy R.I., Zakhary M.M., Maximous D.W., A Mokhtar A., El Dosoky M.I. (2020). Circulating osteocyte-related biomarkers (vitamin D, sclerostin, dickkopf-1), hepcidin, and oxidative stress markers in early breast cancer: Their impact in disease progression and outcome. J. Steroid Biochem. Mol. Biol..

[35] Mariz K., Ingolf J.-B., Daniel H., Teresa N.J., Erich-Franz S. (2015). The Wnt inhibitor dickkopf-1: A link between breast cancer and bone metastases. Clin. Exp. Metastasis.

[36] Kasoha M., Bohle R.M., Seibold A., Gerlinger C., Juhasz-Böss I., Solomayer E.-F. (2018). Dickkopf-1 (Dkk1) protein expression in breast cancer with special reference to bone metastases. Clin. Exp. Metastasis.

[37] Xu X., Zhang M., Xu F., Jiang S. (2020). Wnt signaling in breast cancer: Biological mechanisms, challenges and opportunities. Mol. Cancer.

[38] Geyer F.C., Lacroix-Triki M., Savage K., Arnedos M., Lambros M.B., MacKay A., Natrajan R., Reis-Filho J.S. (2011). Β-Catenin pathway activation in breast cancer is associated with triple-negative phenotype but not with CTNNB1 mutation. Mod. Pathol..

[39] Zhang J., Wei Q., Dong D., Ren L. (2021). The role of TPS, CA125, CA15-3 and CEA in prediction of distant metastasis of breast cancer. Clin. Chim. Acta.

