# Supplementary material for: Retrospective Evaluation of Bone Turnover Markers in Serum for the Prediction of Metastases Development in Breast Cancer Patients: A Cohort Study
Source: Biomedicines. 2024 May 29;12(6):1201. doi: 10.3390/biomedicines12061201 (PMC11201037; doi:10.3390/biomedicines12061201)
Supplement: Supplementary file 1 [file biomedicines-12-01201-s001.zip › biomedicines-2992184-supplementary.pdf]

Protein	Gene	Gene alteration frequency
BAP	ALPL	3%
Carboxyterminal telopeptide of type I collagen (collagen type I)	COL1A1	12%
Tartrate-resistant acid phosphatase type 5 enzyme	ACP5	4%
Dickkopf-related protein 1	DKK1	4%
Sclerostin	SOST	4%
Osteoprotegerin	TNFRSF11B	21%
Receptor activator of nuclear factor kappa-B ligand	TNFSF11	4%

Supplementary Table S1. Summary of human genes for studied proteins along with frequencies of gene alterations in the TCGA cohort published by Ciriello *et al.* (see Methods).
